# “Deep Brain Stimulation of the Ventral Intermediate Nucleus of the Thalamus for Tremor in Polr3a-Related Tremor-ataxia Syndrome: A Two-case Report”

**DOI:** 10.5334/tohm.1000

**Published:** 2025-09-05

**Authors:** Edgar Javier Sánchez-Román, Leonel Villa-Villegas, Roberto Leal-Ortega, Luz Gabriela Lira-Jaime, Francisco Rivas-Ruvalcaba, Karely Díaz-Ramírez, Carlos Eduardo Piña-Avilés, Rodrigo Mercado-Pimentel, Carlos Zúñiga-Ramírez

**Affiliations:** 1 Movement Disorders and Neurodegenerative Diseases Unit, Hospital Civil de Guadalajara “Fray Antonio Alcalde”, Guadalajara, Mexico; 2 Department of Neurology, Hospital Faro del Mayab, Mérida Yucatán, México; 3 Department of Genetics, Hospital Civil de Guadalajara “Fray Antonio Alcalde”, Guadalajara, Mexico; 4 Unidad de Movimientos Anormales y Neurodegeneración de Occidente (UMANO), Hospital San Javier, Guadalajara, México

**Keywords:** POLR3A, spasticity, tremor, ataxia, DBS, Vim, thalamus

## Abstract

**Clinical Vignette:**

RNA polymerase III subunit A (POLR3A) related disorders are a group of heterogeneous diseases with a recessive autosomic inheritance. These disorders manifest with distinct clinical features like ataxia, spasticity, hypodontia, hypogonadism, mental retardation and progressive motor decline.

**Clinical Dilemma:**

POLR3A gene mutation can manifest with parkinsonism, dystonia, ataxia and tremor. Deep brain stimulation (DBS) might be effective for motor symptoms. Choosing the best DBS target is essential for successful treatment.

**Case reports and Clinical Solution:**

Two subjects with a predominant tremorous syndrome due to POLR3A gene mutation with no response to pharmacological treatment underwent DBS at ventral intermediate nuclei (Vim DBS) of thalamus, with significant improvement in tremor.

**Gap in Knowledge:**

Tremor in POLR3A gene mutation could respond to Vim DBS.

## Clinical Vignette and Background

POLR3A-related disorders are a group of diseases with a wide spectrum of manifestations. Despite its rarity, there are some isolated case reports [1,2,3,4,5,6,7,8,9,10,11], case series and cohort studies [12,13,14,15,16,17,18,19,20] reporting that around 3% of cases with spastic paraplegia and cerebellar syndrome are related to this mutation [19].

POLR3A encodes the subunit A of RNA polymerase III (Pol III), responsible for transcription of specific groups of genes and expression of some non-coding RNAs [21, 22]. Mutations of Pol III gene clinically can be seen as a movement disorder with ataxia and tremor as the main features, but also, it can manifest with cognitive impairment, endocrinological, dental and ophthalmic changes [23].

We describe two subjects with a compound heterozygous pathogenic variant at the POLR3A gene, depicting a tremor-ataxia syndrome and right-hand tremor respectively, with striking improvement after Vim DBS.

## Case reports

### Case 1: Male with tremor-ataxia POLR3A Phenotype

A 38-year-old right-handed man with no familial background for neurological diseases started at the age of 13 with hyperkinetic movements in his hands. Head and voice tremors were noticed five years later. Gait and balance disorders began when he was 37 years old.

Neurological assessment depicted head, voice and high amplitude arm tremors. Besides this, global hyporeflexia, predominant left dysmetria, dysdiadochokinesia, and mild ataxic gait were also seen. A brain magnetic resonance image (MRI) was performed with no remarkable findings. Nerve conduction studies showed the presence of a mild axonal sensorimotor polyneuropathy.

Complete genomic sequencing was performed, revealing a compound heterozygous pathogenic variant at the POLR3A gene (NM_007055.3:c.1909+22G>A and NM_007055.3:c1770+1G>A). Segregation studies were performed on his mother, showing a heterozygous variant at the POLR3A gene (NM_007055.3:c1770+1G>A). Unfortunately, segregation analysis could not be done in his father since he died at an early age from traumatic injury.

**Figure 1:**
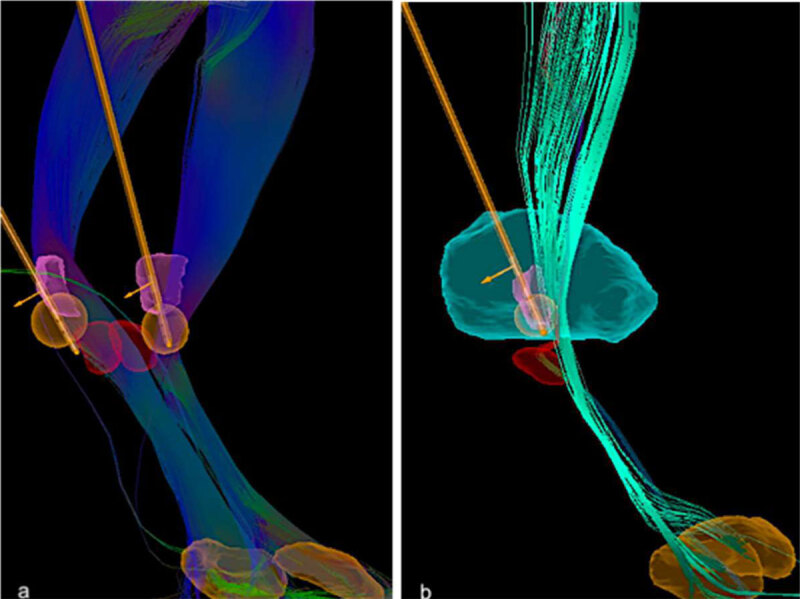
The post- operatory imaging analysis (BrainLab Elements software: fusion, object management, fibertracking, stereotaxy and lead localization modules) showed the leads locations and the volume of tissue activated (VTA) generated according to the most updated stimulation parameters.** a)** Bilateral DBS leads (Medtronic, Activa 3389 model) and the VTAs adjacent to the DRTT at the ventral aspect of both Vim. **b)** Unilateral left DBS directional lead (Boston Scientific, Vercise 2202 model) and the VTA adjacent to the DRRT, at the ventral Vim. *Red nuclei (red), Vim (pink), thalamus (green), DBS leads (orange), VTAs (orange spheres), dentate-rubro-thalamic tracts (DRTT) (turquoise, green and blue)*.

Amantadine, levodopa and topiramate were prescribed with no significant control in tremor. Clozapine provided partial improvement in his tremor; however, this benefit was not helpful for his daily living activities. The Fahn-Tolosa-Marin (TFM) clinical rating scale for tremor score was 91 points. Bilateral Vim DBS was performed, showing great improvement in tremor with no worsening of ataxia (Video 1). Vim DBS has been an effective therapy for tremor, showing adequate control for almost 2 years. The TFM at last visit was 38 points. Current DBS parameters are: –0 –1 –2 +C, amplitude 3 volt (V), pulse width 90 msec and frequency of 200 hertz (Hz) on both sides (Medtronic RC system) (See Figure 1-a).

**Video 1:** **Tremor–ataxia syndrome in a complex POLR3A-related phenotype**. Patient 1 with a predominant tremor–ataxia syndrome, including head and vocal tremor, high-amplitude upper limb tremor, and upper limb ataxia. After bilateral Vim DBS tremor improved remarkably, while ataxia continued to progress.

### Case 2. Female with tremor due to POLR3A

A 38-year-old right-handed woman from Belize with German ancestry, born from nonconsanguineous parents, presented a disabling right-hand tremor. She started at the age of 20 with mild postural and action tremor in both hands, predominantly in the right side.

Her initial diagnosis was essential tremor, and she received oral anti-tremor drugs including propranolol, primidone, levetiracetam and topiramate having significant side effects and showing no improvement. During the following years, tremor increased in severity only in the right side, interfering with basic activities like writing and using of everyday utensils, to the point of no longer using the hand because of disability. She also developed mild difficulties in walking because of “stiffness” of legs, mainly the right.

Her mother was affected by tremor in both hands and one sibling suffered from a chronic gait disorder. There was no history of endocrinological or reproductive disturbances. Her general physical examination was normal.

Neurologic examination disclosed proximal and distal right upper limb tremor present with arms outstretched that worsen to a coarse tremor with arms abducted in “wing beating” posture and finger-nose maneuver; she was unable to handwriting and to mimic feeding herself with a spoon, also spilled the water from the glass when she tried to put in her mouth. The TFM clinical rating scale for tremor score was 56 points (see Video 2, part 1). Brisk deep tendon reflexes were present in all the extremities without other pyramidal signs, also mild spasticity in lower extremities was detected. Rest neurological exam was unremarkable.

**Video 2:** **Tremor in a POLR3A-related phenotype**. Patient 2 presenting with a high-amplitude tremor of the right upper limb, significantly interfering with daily activities. Following unilateral left Vim DBS, tremor improved and remained well controlled at 4 months.

**Figure 2:**
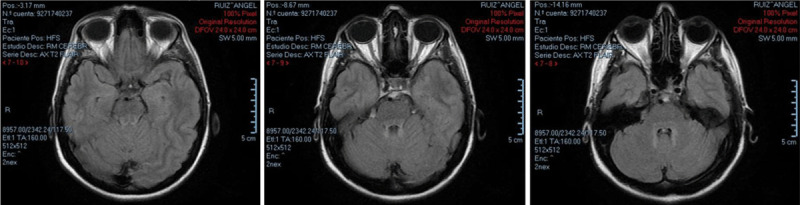
Case 2 brain MRI with bilateral T2/FLAIR hyperintense signal located in the superior cerebellar peduncle, around the IV ventricle, and dentate nuclei.

General blood tests, chemistry, alpha-fetoprotein, vitamins B1, B9 and B12 were normal. Brain MRI showed bilateral T2/FLAIR hyperintense signal located in the superior cerebellar peduncle, around the IV ventricle, and dentate nuclei; no signs of classical hypomyelination or white matter changes, nor cerebellar atrophy were present (See Figure 2).

Given the family history of a mother with upper limb tremor and a sibling with a slowly progressive ataxic gait with spasticity starting in adolescence, along with the presence of a chronic motor-sensitive demyelinating polyneuropathy with axonal degeneration and with similar brain MRI findings, a genetic etiology was considered. Whole genome sequencing revealed a compound heterozygous pathogenic variant at the POLR3A gene (NM_007055.4:c.1740dup and NM_007055.3:c.1909+22G>A).

Due to the disability caused by tremor and poor tolerance to oral pharmacotherapy, she underwent unilateral left Vim DBS. After 4-month post-operative follow-up, she fully regained her handwriting and had minimal symptomatic tremor in the right arm; TFM scale at last visit was 8 points (see Video 2, part 2). DBS settings at that time were: left Vim case positive, contacts 2, 3 and 4 negative, (directional lead, Vercise Gevia^™^, Boston Scientific®) amplitude 3.8 milliamps (mA), pulse width 60 msec, rate 130 Hz (See Figure 1-b).

## Clinical Dilemma

RNA polymerase III (POLR3) is crucial for the synthesis of small, non-coding RNAs including transfer RNAs (tRNAs), 5S ribosomal RNA, 7S RNA, and U6 small nuclear RNA, which are involved in the regulation of transcription, RNA processing, and translation [22, 24]. POLR3 is encoded by POLR3A, POL3B, POLR1C and POLR3K genes. Mutations of these genes cause abnormal tRNA and non-coding RNA transcription in a cell type and growth state dependent manner, and can impact cellular growth, differentiation and apoptosis [21, 24]. POLR3A gene is constitutively expressed in all tissues, with data showing a higher expression in the cerebellum. POLR3A mutation may lead to dysfunction of Pol III, which affects the expression of certain tRNAs that are more abundant in the central nervous system (CNS), thus disrupting protein synthesis [13].

The clinical spectrum of POLR3A-related disorders is highly heterogeneous. However, a consistent feature across the clinical presentations is the presence of movement disorders, including ataxia, tremor, dystonia and spastic paraplegia [1, 14, 16, 25].

To date, at least seven distinct clinical phenotypes have been associated with POLR3A pathogenic variants [10, 26,27,28,29,30,31,32] (see Table 1). Among them, Hypomyelinating Leukodystrophy Type 7 (HLD7) is the most frequently reported condition. Other phenotypes include: Hypodontia, Hypogonadotropic Hypogonadism, and Hypoplasia of the corpus callosum (4H) syndrome; Ataxia, Delayed Dentition, and Hypomyelination (ADDH) syndrome; Tremor-Ataxia with Central Hypomyelination (TACH) syndrome; Leukodystrophy with Oligodontia (LO) syndrome; Hypomyelination with Cerebellar Atrophy and Hypoplasia of the corpus callosum (HCAHC) syndrome, and Wiedemann–Rautenstrauch syndrome.

**Table 1:** Clinical phenotypes of POLR3A-related diseases.


SPECTRUM OF POLR3A-RELATED DISEASE							

CONDITION	HYPOMYELINATING LEUKODYSTROPHY TYPE 7 (HLD-7) [11]	4H LEUKODYSTROPHY SYNDROME [26]	ATAXIA, DELAYED DENTITION, AND HYPOMYELINATION (ADDH) SYNDROME [27]	TREMOR-ATAXIA WITH CENTRAL HYPOMYELINATION (TACH) SYNDROME [28]	LEUKODYSTROPHY WITH OLIGODONTIA (LO) SYNDROME [29]	HYPOMYELINATION WITH CEREBELLAR ATROPHY AND HYPOPLASIA OF THE CORPUS CALLOSUM (HCAHC) SYNDROME [30]	WIEDEMANN-RAUTENSTRAUCH SYNDROME [32]

Age of onset	Infancy or childhood	Childhood, adolescence, or early adulthood	Childhood or adolescence	Infancy or early childhood	Childhood or adolescence	Adolescence or adulthood	Neonatal period

Neurological features	Neurodevelopmental delay Ataxia, tremor, dysarthria, spasticity, dystonia	Neurodevelopmental delay	Dysarthria, hypometric saccades, limb ataxia, dysdiadochokinesia, gait ataxia, intention tremor, pyramidal signs	Neurodevelopmental delay or cognitive decline, dysarthria, limb ataxia, gait ataxia, upper-limb tremor, dystonia, myoclonus, pyramidal signs, nystagmus	Dysarthria Mild limb ataxia Mild intention tremor Lower limb spasticity Pyramidal signs	Dysarthria, ataxia; tremor, dystonia, spasticity, intellectual disability or cognitive decline	Tremor, ataxia, hypertonia

Non-neurological features	Dental abnormalities (absent or delayed eruption of teeth) Endocrine abnormalities (delayed, halted, or absent puberty) Short stature Ocular abnormalities (myopia, optic atrophy, cataracts)	Dental abnormalities (hypodontia) Endocrine abnormalities (hypogonadotropic hypogonadism [delayed puberty])	Dental abnormalities (delayed dentition, abnormal order of teeth eruption, variation in mineralization of permanent teeth, natal teeth) Short stature	Dental abnormalities (hypodontia, delayed tooth eruption, oligodontia, irregular tooth shape or placement) Endocrine abnormalities (hypogonadotropic hypogonadism [delayed puberty]) Short stature Ocular abnormalities (myopia)	Dental abnormalities (delayed dental eruption, oligodontia) Craniofacial anomalies (enophthalmia)	Not described	Obstetrical abnormalities (growth deficiency) Craniofacial anomalies (sparse scalp hair, triangular face, small mouth with thin upper vermillion, pointed chin; other anomalies include prominent scalp veins, wide cranial sutures, ears anomalies) Generalized lipodystrophy Dental abnormalities (natal teeth, hypodontia)

Radiological features	Diffuse hypomyelinations with variable T1 signal intensity; T2 high signal in the white matter of the brain, and relative T2 hypointense signal in the anterolateral nuclei of the thalami, dentate nucleus, globus pallidus, pyramidal tracts of the posterior limb of the internal capsule, and optic radiations; cerebellar atrophy; thinning of the corpus callosum	Diffuse hypomyelination; hypointense signal of the optic radiation, the ventrolateral thalamus, part of the posterior limb of the internal capsule, and the dentate nucleus; Cerebellar atrophy more of the vermis than of the hemispheres; Spinal cord hypomyelination	Hypomyelination of the supratentorial white matter (hyperintense signal on T2); Thin corpus callosum; Cerebellum atrophy (particular in the vermis)	Diffuse supra-tentorial hypomyelination involving periventricular and the subcortical region as well as U-fibers. Thinning of corpus callosum	Diffuse leukodystrophy of the deep white matter of both centrum semi-ovale and cortico-spinal tracts	Lack of myelin throughout the supratentorial cerebral white matter; Thin corpus callosum; cerebral atrophy; mild cerebellar atrophy	Hypomyelination; Polymicrogyria of the perisylvian cortex


The most frequently reported neuroimaging findings include hypomyelination of the centrum semi-ovale, internal capsule, deep white matter, and corpus callosum [31], as well as cerebellar atrophy [33]. Additional structures that may be affected include the putamen, caudate nucleus, and red nucleus [31].

These cases show action tremor (postural and intention) as the most disabling feature, in addition to cerebellar manifestations in case 1. However, in cases where this mutation is seen, spastic ataxia (SPAX) and pyramidal signs are the most prominent features [1, 14, 18, 23]. Case 1 resembles TACH syndrome, nevertheless, imaging study did not show hypomyelination, which is typical of this disorder. In a review carried out by *Sun* et al. [23], there were 31 cases with POLR3A mutations, all of them showed hypomyelination in the internal and external capsules, white matter, corpus callosum and cerebellum [34]. This brain MRI findings were absent in this case, which further expands the heterogeneity of the presentation of this mutation. On the other hand, Case 2 presented with right-hand postural and action tremor, brisk deep tendon reflexes and mild lower limb spasticity without other neurological features. Her brain MRI only depicted unspecific T2 hyperintensities in the superior cerebellar peduncle, around the IV ventricle, and dentate nuclei. Interestingly, given the differing clinical presentation in her sibling, genetic testing was deemed necessary to investigate a potential familial etiology.

Since POLR3A gene mutations manifest predominantly as a movement disorder pharmacological approach might be considered as a first line treatment for tremor, dystonia and parkinsonism [35], nonetheless with a modest and transient response over time [36].

## Clinical Solution

Deep brain stimulation of the globus pallidus has been reported to provide beneficial effects in individuals with POLR3A pathogenic variants presenting with parkinsonism and dystonia [35, 36]. Additionally, bilateral Vim DBS has proven effective in treating various tremor-predominant conditions, including essential tremor, tremor-dominant Parkinson’s disease, tremor-dystonia, and Holmes tremor [37,38,39]. Based on this prior evidence and considering that tremor was the predominant symptom in both of our cases, Vim DBS was performed, resulting in an improvement in both tremor frequency and amplitude.

## Gap in Knowledge and Conclusion

Diagnosis approach of POLR3 HLD needs considerations of clinical features, findings on MRI as well as the determination of a pathogenic variant. However, given a wide spectrum of clinical manifestations and involvement of multiple systems, diagnosis is challenging. We remark that genetic tests are mandatory not only for an accurate diagnosis but to guide the therapy approach as DBS exhibited a significant control of tremor and other movement disorders in POLR3A diseases.

These subjects showed a favorable response to Vim DBS; however, given the short follow-up period in case 2, these results should be interpreted with caution, despite the positive clinical response observed so far.

Since there are only a few reported cases of patients undergoing DBS, (especially with Vim DBS), our case expands surgical treatment possibilities.
